# Delay-Tolerant Distributed Inference in Tracking Networks

**DOI:** 10.3390/s21175747

**Published:** 2021-08-26

**Authors:** Mohammadreza Alimadadi, Milica Stojanovic, Pau Closas

**Affiliations:** Department of Electrical and Computer Engineering, Northeastern University, Boston, MA 02115, USA; millitsa@ece.neu.edu (M.S.); closas@northeastern.edu (P.C.)

**Keywords:** data fusion, statistical inference, distributed learning, partial estimators, Kalman filter, object tracking, wireless sensor network, random access, delay

## Abstract

This paper discusses asynchronous distributed inference in object tracking. Unlike many studies, which assume that the delay in communication between partial estimators and the central station is negligible, our study focuses on the problem of asynchronous distributed inference in the presence of delays. We introduce an efficient data fusion method for combining the distributed estimates, where delay in communications is not negligible. To overcome the delay, predictions are made for the state of the system based on the most current available information from partial estimators. Simulation results show the efficacy of the methods proposed.

## 1. Introduction

Wireless sensor networks (WSNs) are composed of a number of resource-constrained sensor nodes that are capable of perceiving the environment, collecting, processing, and exchanging data. WSNs have numerous applications, including battlefield surveillance, data gathering, agent actuation, health-care, smart cities, and object tracking [1,2].

One of the main applications of WSNs is object tracking, which has been studied extensively in the past few decades, with numerous tracking algorithms being proposed [3,4]. However, object tracking is still a very challenging problem [5]. The primary objective of the tracking algorithms is to maintain accuracy while reducing the communication overhead and energy consumption [6,7,8,9,10].

In object tracking, parameters of the model often need to be inferred from a set of measurements. Performing statistical inference in these situations imposes computational/memory/storage constraints that make global estimation impractical [11]. Moreover, due to the nature of many applications, the data are often collected in a distributed manner. As a result, it has become extremely important to develop algorithms that are both advanced enough to capture the complexity of the modern data and scalable enough to process large datasets in a parallelized or fully decentralized fashion. A common solution is to divide the data among multiple partial estimators (PEs) and to combine their estimates together to form a final one. The goal in doing so is to find an optimal combination that achieves a performance as close as possible to that of a global estimator that has access to all the information.

Data fusion of PEs has been studied in different contexts including signal processing, digital communications, and control and sensor networks [12,13,14]. A general procedure to combine estimators has been proposed in [15] for the case where there are multiple parameters in the system. Time series forecasting [16], distributed estimation in wireless sensor networks [17,18,19], optimal linear fusion for multi-dimensional cases [20], distributed fusion by adapting methods for graphical models [21], and different consensus, gossip, or diffusion algorithms [22,23,24] are some of the approaches proposed. Our previous work addressed distributed tracking in underwater acoustic sensor networks [25,26]. We designed a scalable method for large area coverage, in which multiple fusion centers, each overseeing sensors within a smaller footprint, exchange local tracking information to reach a consensus. In the consensus-oriented distributed filtering mode, sensors are arranged in groups, and each group has a PE that runs an independent filter [27,28] while exchanging information with its neighbors iteratively. As a result, PEs can reach a consensus [22], with the benefits of network cooperation in terms of improved performance, better robustness, and resilience to failure.

More broadly, today, there is a prevalence of distributed networked sensors, such as drones, security cameras, and Internet of Things (IoT) devices, that perform surveillance, target tracking, and mapping from high-dimensional streaming inputs, including video. These distributed devices typically aggregate their information at a centralized node or a data center for sensing tasks such as computationally intensive mapping, computer vision tasks, or querying frequently updated databases such as Google Images. The sensing itself is increasingly performed using computationally intensive neural networks (often pre-trained) that can scalably run in cloud environments.

Decentralized architectures rely on communication links between cooperating platforms. In doing so, the number of messages that each platform sends or receives is independent of the total number of platforms in the system. This property ensures scalability to distributed systems with (almost) any number of platforms. Such approaches have their own limitations. In some applications, PEs are either the cores in a multi-core worker or machines in a multiple-machine setup. In either case, communications occur over a wired platform and the delay is considered negligible. This is not a reasonable assumption in other scenarios where wireless communication may induce a delay, be it because of long transmission distance (satellite or deep space), low speed of propagation (sound in water or air), or buffering en route. Moreover, unlike regular distributed fusion, synchronization is not easy to achieve in some situations. Consequently, there is a need to address the effect of delay and asynchronous communication.

There have been a number of studies in this area; however, they do not offer solutions that would compensate for the delay and allow the system to operate seamlessly. Instead, they identify the conditions under which the system can tolerate a certain delay. For example, Ref. [27] offers a threshold for delay under which the agents (or partial estimators) can reach the consensus.

In this paper, we study the problem of distributed inference in object tracking networks in the presence of delays. We introduce an efficient data fusion method for combining the distributed estimates, where communication delay is taken into account. We concentrate on the minimum mean squared error (MMSE) global estimator, but the developed framework is general and can be used to combine other types of unbiased partial estimators.

The rest of the paper is organized as follows. Section 2 presents the system model and problem definition. In Section 3, the concept of fusion with overwriting is introduced. Section 4 discusses the problem of delay along with our proposed solution.

Other applications are discussed in Section 5. Numerical results are shown in Section 6. Finally, we provide concluding remarks in Section 7.

## 2. System Model and Problem Definition

In this paper, we consider a scenario similar to the one introduced in [25,29], where a number of objects are moving inside an observation area. Each of the objects broadcasts a signal of a certain amplitude. The signal attenuates as it propagates away from the object. The attenuation follows a signature function, which is assumed to be known. Examples of attenuation functions include exponential decay and distance-squared decay.

We assume that a number of sensor nodes are distributed across the observation area. Each sensor node performs measurements, encodes the obtained value into a digital data packet, adds its ID, and transmits the packet to a fusion center (FC). The measurements may occur at random instants in time.

The FC collects the packets transmitted in one time interval of duration *T*. It is assumed that the interval is selected to be short enough so that the objects’ locations can be considered fixed over it. Some of the transmitted packets may be dropped because of collisions and noise. Packets that are received correctly are then used as an input to the tracking algorithm to estimate the location of the objects. We define the state of the system at time t=kT as

(1) $$x_{k}={\left[x_{k}\left(1\right)\;x_{k}\left(2\right)\;\ldots \;x_{k}\left(M\right)\right]}^{⊤}\in R^{M}$$

The states can represent the location/velocity/acceleration of moving objects, for instance. The time *T* is the updating interval. The general Markovian non-linear state transition model is given by

(2) $$x_{k}=f\left(x_{k-1}\right)+\nu_{k}$$

where f(·) is a known and potentially non-linear function of the state and $\nu_{k}$ is the process noise. The goal is to estimate the state of the system, given a set of observations.

We assume that there are *L* partial estimators in the system. Each PE estimates the state of the system based on the set of observations available to it,

(3) $$y_{k}^{\left(\ell \right)}=h^{\left(\ell \right)}\left(x_{k}\right)+\eta_{k}^{\left(\ell \right)},\;\;\ell =1,\ldots ,L$$

where $h^{\left(\ell \right)}\left(\cdot \right)$ is the *ℓ*-th measurement function.

At time interval *k* the inputs to the *ℓ*-th PE are the partial prior state estimate ${\hat{x}}_{k^{-}}^{\ell }$, the partial prior covariance matrix ${\hat{C}}_{k^{-}}^{\left(\ell \right)}$, and the observation vector $y_{k}^{\left(\ell \right)}$. Based on these inputs, the partial estimator generates ${\hat{x}}_{k}^{\ell }$ and ${\hat{C}}_{k}^{\left(\ell \right)}$ as

(4) $$\left[{\hat{x}}_{k}^{\left(\ell \right)},{\hat{C}}_{k}^{\left(\ell \right)}\right]=P\left({\hat{x}}_{k^{-}}^{\left(\ell \right)},{\hat{C}}_{k^{-}}^{\left(\ell \right)},y_{k}^{\left(\ell \right)}\right)$$

where P(·) is the partial estimation function. Examples of partial estimation methods are gradient descent and Kalman filtering [30]. We assume that each PE has an initial estimate ${\hat{x}}_{1^{-}}^{\left(\ell \right)}$ and an initial covariance matrix ${\hat{C}}_{1^{-}}^{\left(\ell \right)}$.

**Figure 1:**
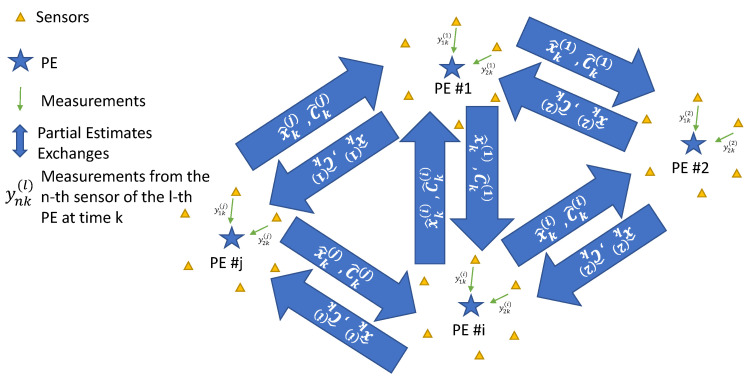
The overall schematic of the system.

Figure 1 depicts the overall schematic of the system.

## 3. Data Fusion

At the end of the updating interval, each PE has an estimate of the state vector. The PEs exchange their partial estimates in order to fuse the information, i.e., reach a consensus on the state of the system. The final estimate of the state of the system at time *k* is

(5) $$\left[{\hat{x}}_{k}^{F},{\hat{C}}_{k}^{F}\right]=F\left({\hat{x}}_{k}^{\left(1\right)},\ldots ,{\hat{x}}_{k}^{\left(L\right)},{\hat{C}}_{k}^{\left(1\right)},\ldots ,{\hat{C}}_{k}^{\left(L\right)}\right)$$

where F(·) is the fusion function. A fusion method thus accepts as inputs ${\hat{x}}_{k}^{\ell }$ and ${\hat{C}}_{k}^{\left(\ell \right)}$ for all ℓ=1,…,L and combines them into the outputs ${\hat{x}}_{k}^{F}$ and ${\hat{C}}_{k}^{F}$, respectively. The linear minimum mean square error estimator (LMMSE) is an example of a fusion function [31].

### Overwriting

Upon completion of the fusion step, a new round of estimation begins. This round takes as the input the estimates ${\hat{x}}_{{\left(k+1\right)}^{-}}^{\left(\ell \right)}$ and the covariances ${\hat{C}}_{{\left(k+1\right)}^{-}}^{\left(\ell \right)}$, which are set to the current values produced by the corresponding partial estimator,

(6) $${\hat{x}}_{{\left(k+1\right)}^{-}}^{\left(\ell \right)}={\hat{x}}_{k}^{\left(\ell \right)}\;\;\mathrm{for}\;\;\ell =1,\ldots ,L$$

and

(7) $${\hat{C}}_{{\left(k+1\right)}^{-}}^{\left(\ell \right)}={\hat{C}}_{k}^{\left(\ell \right)}\;\;\mathrm{for}\;\;\ell =1,\ldots ,L$$

Alternatively, the partial estimates can be overwritten with the final estimate,

(8) $${\hat{x}}_{{\left(k+1\right)}^{-}}^{\left(\ell \right)}={\hat{x}}_{k}^{F}\;\;\mathrm{for}\;\;\ell =1,\ldots ,L$$

and the covariance matrix can be overwritten as well:

(9) $${\hat{C}}_{{\left(k+1\right)}^{-}}^{\left(\ell \right)}={\hat{C}}_{k}^{F}\;\;\mathrm{for}\;\;\ell =1,\ldots ,L$$

Since the final estimate is a result of a fusion of the partial estimates, one expects overwriting to offer an improvement in performance. To assess the benefits of overwriting, several definitions are in order.

**Definition** **1.** *An unbiased estimation method P(·,·,y) is said to be* plain *if for the same y, two input covariances $C_{k_{1}^{-}}≻C_{k_{2}^{-}}$ yield output covariances such that $C_{k_{1}}≻C_{k_{2}}$. For two square matrices A and B, we write A≻B if A−B is positive definite.*

An example of a plain estimation method is Kalman filtering. For the special case of one-dimentional state-space model, the variance provided by the Kalman filter is $C_{k}=\frac{\left(A^{2}C_{k^{-}}+Q\right)R}{\left(A^{2}C_{k^{-}}+Q\right)H^{2}+R},$ which is a plain function of $C_{k^{-}}$. Here, *A* and *H* are the state transition parameter and the observation parameter, respectively, and *Q* and *R* are the variances of the process noise and measurement noise, both modeled as white and Gaussian.

**Definition** **2.** *An unbiased fusion method F(·,·) is called proper if $C_{k}^{F}⪯C_{k}^{\left(\ell \right)}$ for all ℓ and k.*

For example, LMMSE is a proper fusion method. A proof of this fact can be found in Appendix A.

With these definitions, the following theorem states the conditions under which the overwriting process leads to improvement in the performance of the system.

**Theorem** **1.** *In an (estimation, fusion) pair, if the estimation method P is plain and the fusion method F is proper, then at each time, overwriting ((8) and (9)) has better performance in the mean-squared error sense than no overwriting ((6) and (7)).*

**Figure 2:**
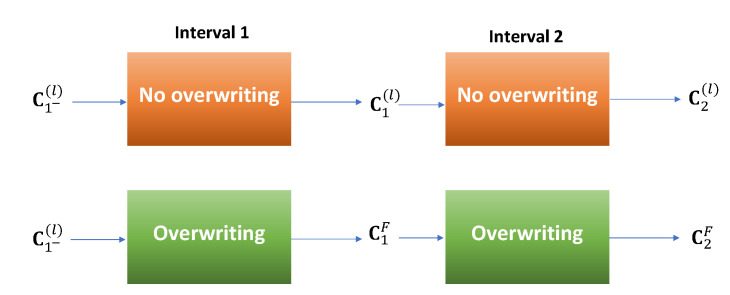
No overwriting (**top**) vs. overwriting (**bottom**) for the first two iterations.

**Proof.**  Starting with the same $C_{1^{-}}^{\left(\ell \right)}$ and $y_{1}^{\left(\ell \right)}$ as the input to both methods, the output covariance of the overwriting method is $C_{1}^{F}$, while the output without overwriting is $C_{1}^{\left(\ell \right)}$ for ℓ=1,…,L. Figure 2 depicts the first two intervals. Because the fusion method is proper, we have that $C_{1}^{F}⪯C_{1}^{\left(\ell \right)}$ for all *ℓ*. These values are the inputs to the second interval. Because the estimation method is plain, $C_{2}^{F}⪯C_{2}^{\left(\ell \right)}$ for all ℓ=1,…,L. These, in turn, are the inputs to the next interval and so on. Please note that since all the methods here are unbiased, it is sufficient to compare only the covariance matrices and not the actual estimates. With the same line of reasoning, it can be easily shown that the same conclusion holds for the other intervals. Therefore, $C_{k}^{F}⪯C_{k}^{\left(\ell \right)}$ for all *k* and the proof is complete. □

As we saw from the example above, many of the estimation/fusion combinations such as the Kalman filter/LMMSE pair in object tracking fall into the category of methods that have better performance with overwriting.

## 4. Data Fusion in the Presence of Delay

To date, the assumption was that there is no delay in the process of exchanging the partial estimates. In practice, however, there is typically some delay that needs to be taken into account. We have shown that overwriting improves the performance of the system, but we did so assuming no delay. The question thus arises as to how sensitive the overwriting process is to a delay, and whether adjustments need to be made when a non-negligible delay is present. Moreover, the overwriting procedure as we have it at the moment assumes synchronization between PEs. Synchronization is difficult to achieve in many practical scenarios, and asynchronous operation may be the only option [29]. The fact that the computational power improves much faster than communication speeds in the modern digital world requires a closer look at the problem of delay in distributed inference.

In overwriting, PEs need the final estimate before they can run the next iteration. However, there might be a delay between the time when the partial estimates are calculated and the time when all PEs receive the final estimate. In this situation, fusion should occur, given the most recent estimates of the other PEs.

At the *k*-th updating interval, the *ℓ*-th PE uses the measurements received during this interval and generates the partial estimate ${\hat{x}}_{k}^{\ell }$ and the covariance matrix ${\hat{C}}_{k}^{\left(\ell \right)}$. The PEs then exchange the information. We assume that it takes $T_{i,j}$ seconds (or $D_{i,j}$ updating intervals) for the partial estimate of the *i*-th PE to reach the *j*-th PE. The goal is to fuse the delayed partial estimates together and produce the final local estimate. Note that one PE’s final estimate may now differ from another’s, which is why we refer to it as the final local estimate.

One way to address the issue of delay is to have each PE wait for the feedback from other PEs and then perform fusion. In this approach, a PE collects the observations during *T*, updates its local estimate using what existing final state estimate it has, and sends that local estimate to other PEs. It then waits for $T_{d}=\max\left(T_{i,j}\right)$. Once all the partial estimates have arrived at the end of the waiting interval, the local PE fuses them with its own. As the local estimate is delayed by the same amount as the neighbors’ estimates, there is no timing discrepancy. The local PE thus has an accurate information about the state of the system $T_{d}$ seconds ago. This information constitutes its final estimate, which will be used at the end of the next updating interval that starts immediately upon fusion. The cycle then repeats: observations are collected during the next *T*, local estimate is updated and sent to other PEs, feedback is received after $T_{d}$ seconds, and fusion is performed.

The duration of the full cycle is now $T+T_{d}$, and hence the system is updated as often. In other words, estimates are available only every $T+T_{d}$ seconds. The update rate is thus reduced compared to the case of no delay (D=0). However, the estimation accuracy is not affected, other than by the fact that the system is observed less frequently. One last point to note is that in order to implement the process, the communication between PEs needs to be synchronous [25].

### Soft Fusion with Predict and Go Method (SoftPG)

While the above procedure may be well suited to some applications, in most other applications, synchronization is not possible and asynchronous performance is the only option. Moreover, waiting incurs a waste of time, during which information is lost. Therefore, we propose a prediction-based method to seamlessly combine estimates and move forward without waiting. The estimates should be updated as often as possible, so as to reflect the current state, not the delayed state.

In this section, we introduce soft fusion with a method termed Predict and Go (SoftPG) which addresses the issue of delay in asynchronous distributed inference. Specifically, each PE will now continue to compute partial estimates every *T* seconds, but at the time when fusion is performed, the information available from the neighboring PEs will be outdated. SoftPG will take this fact into account by first turning the estimates available from other PEs into $D_{i,j}$-step predictions. These predictions will then be used in fusion. The fusion algorithms thus remain the same, except that they operate with predictions made on the partial estimates and their corresponding covariances. Once the fusion is complete, each PE sends its new information out to other PEs. The process thus continues in cycles of duration *T*. The estimates now reflect the current state of the system, and the only effect of delay is through the prediction error.

In SoftPG, the final estimate of the *ℓ*-th PE is given by

(10) $${\hat{x}}_{k}^{\left(\ell ,F\right)}=F\left({\left\{{\hat{\hat{x}}}_{k}^{\left(\ell ,i\right)}\right\}}_{i=1}^{L},{\left\{{\hat{\hat{C}}}_{k}^{\left(\ell ,i\right)}\right\}}_{i=1}^{L}\right)$$

where A is the state transition matrix in (2). The LMMSE fusion is now specified by

(11) $${\hat{x}}_{k}^{\left(\ell ,F\right)}={\left[\sum_{i=1}^{L}{\left({\hat{\hat{C}}}_{k}^{\left(\ell ,i\right)}\right)}^{-1}\right]}^{-1}\left(\sum_{i=1}^{L}{\left({\hat{\hat{C}}}_{k}^{\left(\ell ,i\right)}\right)}^{-1}{\hat{\hat{x}}}_{k}^{\left(\ell ,i\right)}\right)$$

**Figure 3:**
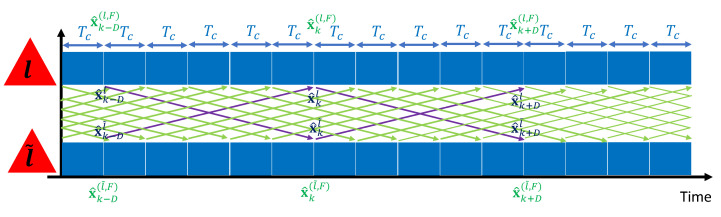
Soft Fusion with Predict-and-Go principle. *ℓ* and $\tilde{\ell }$ are the indices of two partial estimators.

Figure 3 illustrates the process of SoftPG. *ℓ* and $\tilde{\ell }$ are the indices of two partial estimators. Note that communication between the PEs no longer needs to be synchronized. This is an important feature of SoftPG, as it caters to all systems in which synchronization is an issue.

## 5. Other Applications

Distributed inference is closely related to many problems including distributed optimization, social learning, federated learning, distributed filtering, etc. Mathematically, these seemingly different concepts can be considered similar enough to be tackled with the same approach. Below, we illustrate two examples.

In neural network training, the observations are the samples in the training set. The estimation method is back-propagation with gradient descent. In object tracking, the observations are the sensory measurements, and the estimation method is the Kalman filter (or any other method for that matter).

In a linear state-space model, the state of the system evolves as

(12) $$f\left(k,x_{k-1},u_{k}\right)=Ax_{k-1}+q_{k}$$

where A is the state transition matrix and $q_{k}$ is the process noise, which is modeled as zero-mean Gaussian with covariance Q. The observation vector, $y_{k}^{\left(\ell \right)}$, contains the measurements obtained by the sensor nodes. In this case, ${\hat{x}}_{1^{-}}^{\left(\ell \right)}$ is the initial estimate of the state vector. Other examples of applications include distributed optimization.

## 6. Numerical Results

In this section, we evaluate the performance of the proposed methods through numerical simulation. The results in this sections are obtained by averaging over 300 iterations, each 200 time intervals in length. Without loss of generality, we assume that the delays between all the PEs are the same. In other words, $T_{i,j}=DT$ for all *i* and j≠i, where *D* is called the normalized delay. The performance is measured using mean-squared error between the actual state of the system and the estimated one. No specific assumption is made regarding the quantities that the state vector represents. Each entry can be the location/velocity of an object but it may also represent other quantities/scenarios.

**Figure 4:**
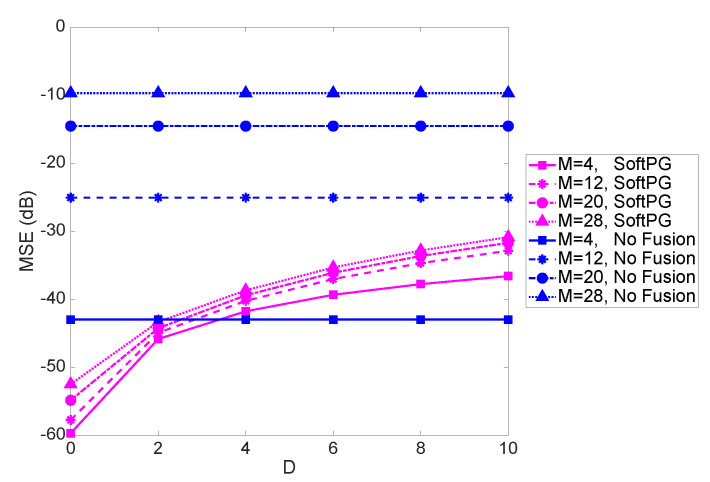
MSE as a function of the normalized delay for different values of *M*, the size of the state vector. The No Fusion curves are shown as the benchmark. Number of observations per PE is 9.

Figure 4 shows the system performance in terms of the mean squared error (MSE) of the delayed estimate as a function of the normalized delay *D*. The number of observations per PE (OPE) is the size of the partial observation vector $y_{k}^{\left(\ell \right)}$ and is fixed at 9. Two sets of curves are shown, one referring to soft fusion with Predict and Go technique, and another, labeled “No Fusion," referring to the scenario where each PE works independently and there is no exchange of information between the PEs. Each set contains four curves corresponding to the varying size of the state vector. We note that focusing on a given *M*, the benefits of fusion are evident up to a certain value of delay. After that, prediction becomes obsolete, and exchanging no information becomes better than exchanging severely outdated information. While that is to be expected, the important observation to make is that that the range of delays where fusion offers benefits is quite considerable. Consider, for example, an object tracking scenario with the linear state-space model,

(13) $$f\left(k,x_{k-1}\right)=Ax_{k-1}+\nu_{k}$$

and T=1 s, where $\nu_{k}$ is the process noise. In this case, the state vector, $x_{k-1}$ is defined as

(14) $$x_{k-1}=\left[x_{1}\;x_{2}\cdots \right]$$

where $x_{i}=\left[x_{i}\;y_{i}\;v_{x_{i}}\;v_{y_{i}}\right]$ is the state vector for the *i*-th object and includes the locations as well as velocities of the *i*-th object in x and y directions. With M=28, corresponding to weven objects being tracked in a 2D space, we see that D=5 or 10 would be well within the tolerable range. This is an encouraging observation as it shows that the effect of delay is not detrimental in a considerable range of practical values. For example, for the special case of object tracking with T=1 s, $D_{i,j}=5$ or 10 would be well within the range of practical delays. As we see in the figure, the difference between the performance of SoftPG and the No Fusion case increases with *M*. This clearly shows the effectiveness of the proposed delay-tolerant method.

**Figure 5:**
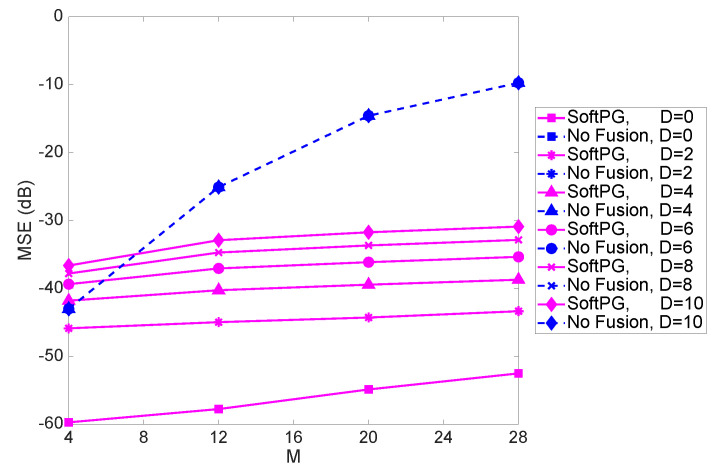
MSE as a function of *M*, the size of the state vector, for different values of delay. Number of observations per PE is 9.

Figure 5 shows the MSE as a function of the size of the state vector, *M*, for different values of delay. For higher values of *M*, the difference between the performance of the SoftPG and the No Fusion benchmark increases. Moreover, one can conclude that with fusion there is less sensitivity to M.

**Figure 6:**
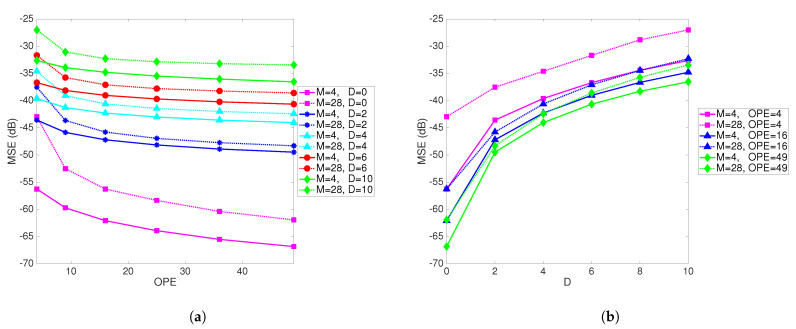
(**a**) MSE as a function of the number of observations per PE (OPE) for different *M* and different delays. Number of PEs is fixed at 9. (**b**) MSE as a function of delay for different *M* and the number of observations per PE. Number of PEs is fixed at 9.

Figure 6a shows MSE as a function of the number of observations per PE for different values of *M* and different delays. The number of PEs is fixed at 9. Figure 6b shows the MSE as a function of delay for different values of *M* and the number of observations per PE. When the number of observations per PE is increased, the performance improves, as one would expect. This result again illustrates the effect of delay and provides a design guideline. For a pre-specified value of a tolerable MSE, and a known delay, it points to the necessary number of observations per PE.

**Figure 7:**
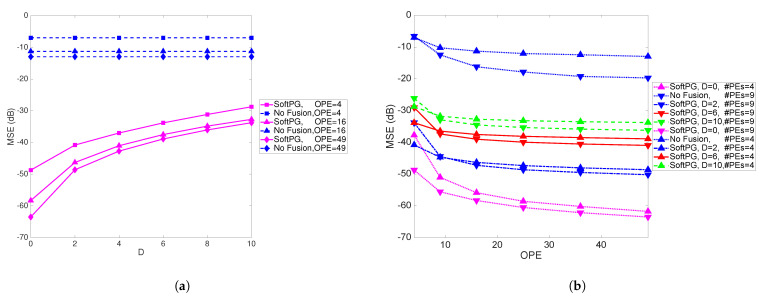
(**a**) MSE as a function of delay for different number of observations per PE (OPE). The size of the state vectors is M=20. (**b**) MSE as a function of the number of observations per PE. Two cases are shown, one with 4 PEs and another with 9. M=20.

In Figure 7a the performance of the system is illustrated as a function of the delay for varying number of observations per PE. Clearly, the difference between the performance of the SoftPG method and the No Fusion benchmark is quite large. Figure 7b shows the MSE as a function of the number of observations per PE per iteration for two numbers of PEs. As expected, for a specific number of observations per PE, by increasing the number of PEs, the performance improves.

## 7. Conclusions

The effect of delay in distributed inference is studied for tracking networks. A delay-tolerant data fusion method is introduced to combine the partial estimates. The method can be used in a wide range of applications where delay is not negligible. In soft fusion with the Predict and Go method, a partial estimator constantly updates its local estimate based on the latest observations and fuses it with the most current estimate available. Synchronization between the partial estimators is not required, only the knowledge of the delay at each PE. The method has a stable performance even for large delays. State overwriting is introduced as a way to improve the performance of the system. Through this process, the fused estimate is used to overwrite all local estimates. Simulation results show the effectiveness of the approach proposed and the substantial improvement with respect to the case where no fusion is used.

## Appendix A

Here we prove that LMMSE is a proper fusion method. To this end, we prove that ${\hat{C}}_{k}^{F}$ is smaller in the positive semi-definite (PSD) sense than all of ${\hat{C}}_{k}^{\left(\ell \right)}$ for ℓ=1,…,L.

$${\hat{C}}_{k}^{F}=E\left[{\hat{x}}_{k}^{LMMSE}{\hat{x}}_{k}^{LMMSE^{⊤}}\right]-E\left[{\hat{x}}_{k}^{LMMSE}\right]E{\left[{\hat{x}}_{k}^{LMMSE}\right]}^{⊤}$$

$$=E\left\{\left[{\left[\sum_{\ell =1}^{L}{\left({\hat{C}}_{k}^{\left(\ell \right)}\right)}^{-1}\right]}^{-1}\sum_{i=1}^{L}{\left({\hat{C}}_{k}^{\left(i\right)}\right)}^{-1}{\hat{x}}_{k}^{i}\right]\;{\left[{\left[\sum_{\ell =1}^{L}{\left({\hat{C}}_{k}^{\left(\ell \right)}\right)}^{-1}\right]}^{-1}\sum_{j=1}^{L}{\left({\hat{C}}_{k}^{\left(j\right)}\right)}^{-1}{\hat{x}}_{k}^{j}\right]}^{⊤}\right\}$$

$$-E\left[{\left[\sum_{\ell =1}^{L}{\left({\hat{C}}_{k}^{\left(\ell \right)}\right)}^{-1}\right]}^{-1}\sum_{i=1}^{L}{\left({\hat{C}}_{k}^{\left(i\right)}\right)}^{-1}{\hat{x}}_{k}^{i}\right]E{\left[{\left[\sum_{\ell =1}^{L}{\left({\hat{C}}_{k}^{\left(\ell \right)}\right)}^{-1}\right]}^{-1}\sum_{j=1}^{L}{\left({\hat{C}}_{k}^{\left(i\right)}\right)}^{-1}{\hat{x}}_{k}^{j}\right]}^{⊤}=$$

$$E\left\{\left[{\left[\sum_{\ell =1}^{L}{\left({\hat{C}}_{k}^{\left(\ell \right)}\right)}^{-1}\right]}^{-1}\sum_{j=1}^{L}{\left({\hat{C}}_{k}^{\left(i\right)}\right)}^{-1}{\hat{x}}_{k}^{i}\right]\;{\left[{\sum_{j=1}^{L}{{\hat{x}}_{k}^{j^{⊤}}\left({\hat{C}}_{k}^{\left(j\right)}\right)}^{-T}\left[\sum_{\ell =1}^{L}{\left({\hat{C}}_{k}^{\left(\ell \right)}\right)}^{-1}\right]}^{-T}\right]}^{\;}\right\}-$$

$$\left[{\left[\sum_{\ell =1}^{L}{\left({\hat{C}}_{k}^{\left(\ell \right)}\right)}^{-1}\right]}^{-1}\sum_{i=1}^{L}{\left({\hat{C}}_{k}^{\left(i\right)}\right)}^{-1}E\left({\hat{x}}_{k}^{i}\right)\right]{\left[{\left[\sum_{\ell =1}^{L}{\left({\hat{C}}_{k}^{\left(\ell \right)}\right)}^{-1}\right]}^{-1}\sum_{j=1}^{L}{\left({\hat{C}}_{k}^{\left(j\right)}\right)}^{-1}E\left({\hat{x}}_{k}^{j}\right)\right]}^{⊤}=$$

$$E\left\{\left[{\left[\sum_{\ell =1}^{L}{\left({\hat{C}}_{k}^{\left(\ell \right)}\right)}^{-1}\right]}^{-1}\sum_{i=1}^{L}{\left({\hat{C}}_{k}^{\left(i\right)}\right)}^{-1}{\hat{x}}_{k}^{i}{\sum_{j=1}^{L}{{\hat{x}}_{k}^{j^{⊤}}\left({\hat{C}}_{k}^{\left(j\right)}\right)}^{-T}\left[\sum_{\ell =1}^{L}{\left({\hat{C}}_{k}^{\left(\ell \right)}\right)}^{-1}\right]}^{-T}\right]\;\right\}-$$

$$\left[{\left[\sum_{\ell =1}^{L}{\left({\hat{C}}_{k}^{\left(\ell \right)}\right)}^{-1}\right]}^{-1}\sum_{j=1}^{L}{\left({\hat{C}}_{k}^{\left(i\right)}\right)}^{-1}E\left({\hat{x}}_{k}^{i}\right)\right]{\left[{\sum_{j=1}^{L}{E{\left({\hat{x}}_{k}^{j}\right)}^{⊤}\left({\hat{C}}_{k}^{\left(j\right)}\right)}^{-T}\left[\sum_{\ell =1}^{L}{\left({\hat{C}}_{k}^{\left(\ell \right)}\right)}^{-1}\right]}^{-T}\right]}^{\;}=$$

$$E\left\{\left[{\left[\sum_{\ell =1}^{L}{\left({\hat{C}}_{k}^{\left(\ell \right)}\right)}^{-1}\right]}^{-1}\sum_{i=1}^{L}\;{\sum_{j=1}^{L}{\left({\hat{C}}_{k}^{\left(i\right)}\right)}^{-1}{\hat{x}}_{k}^{i}\;{{\hat{x}}_{k}^{j^{⊤}}\left({\hat{C}}_{k}^{\left(j\right)}\right)}^{-T}\left[\sum_{\ell =1}^{L}{\left({\hat{C}}_{k}^{\left(\ell \right)}\right)}^{-1}\right]}^{-T}\right]\;\right\}-$$

$$\left[{\left[\sum_{\ell =1}^{L}{\left({\hat{C}}_{k}^{\left(\ell \right)}\right)}^{-1}\right]}^{-1}\sum_{i=1}^{L}{\left({\hat{C}}_{k}^{\left(i\right)}\right)}^{-1}E\left({\hat{x}}_{k}^{i}\right){\sum_{j=1}^{L}{E{\left({\hat{x}}_{k}^{j}\right)}^{⊤}\left({\hat{C}}_{k}^{\left(j\right)}\right)}^{-T}\left[\sum_{\ell =1}^{L}{\left({\hat{C}}_{k}^{\left(\ell \right)}\right)}^{-1}\right]}^{-T}\right]{=}^{\;}$$

$${\left[\sum_{\ell =1}^{L}{\left({\hat{C}}_{k}^{\left(\ell \right)}\right)}^{-1}\right]}^{-1}\sum_{i=1}^{L}\;{\sum_{j=1}^{L}{\left({\hat{C}}_{k}^{\left(i\right)}\right)}^{-1}E({\hat{x}}_{k}^{i}\;{{\hat{x}}_{k}^{j^{⊤}})\;\left({\hat{C}}_{k}^{\left(j\right)}\right)}^{-T}\left[\sum_{\ell =1}^{L}{\left({\hat{C}}_{k}^{\left(\ell \right)}\right)}^{-1}\right]}^{-T}-$$

$${\left[\sum_{\ell =1}^{L}{\left({\hat{C}}_{k}^{\left(\ell \right)}\right)}^{-1}\right]}^{-1}\sum_{i=1}^{L}\;{\sum_{j=1}^{L}{{\left({\hat{C}}_{k}^{\left(i\right)}\right)}^{-1}E\left({\hat{x}}_{k}^{i}\right)\;E{\left({\hat{x}}_{k}^{j}\right)}^{⊤}\left({\hat{C}}_{k}^{\left(j\right)}\right)}^{-T}\left[\sum_{\ell =1}^{L}{\left({\hat{C}}_{k}^{\left(\ell \right)}\right)}^{-1}\right]}^{-T}=$$

$${\left[\sum_{\ell =1}^{L}{\left({\hat{C}}_{k}^{\left(\ell \right)}\right)}^{-1}\right]}^{-1}\sum_{i=1}^{L}\;{\sum_{j=1}^{L}{\left({\hat{C}}_{k}^{\left(i\right)}\right)}^{-1}[E({\hat{x}}_{k}^{i}\;{{\hat{x}}_{k}^{j^{⊤}})-E\left({\hat{x}}_{k}^{i}\right)\;E{\left({\hat{x}}_{k}^{j}\right)}^{⊤}]\;\;\;\left({\hat{C}}_{k}^{\left(j\right)}\right)}^{-T}\left[\sum_{\ell =1}^{L}{\left({\hat{C}}_{k}^{\left(\ell \right)}\right)}^{-1}\right]}^{-T}=$$

$${\left[\sum_{\ell =1}^{L}{\left({\hat{C}}_{k}^{\left(\ell \right)}\right)}^{-1}\right]}^{-1}\sum_{i=1}^{L}{\left({\hat{C}}_{k}^{\left(i\right)}\right)}^{-1}[E({\hat{x}}_{k}^{i}\;{{\hat{x}}_{k}^{i^{⊤}})-E\left({\hat{x}}_{k}^{i}\right)\;E{\left({\hat{x}}_{k}^{i}\right)}^{⊤}]\;\;\;\left({\hat{C}}_{k}^{\left(i\right)}\right)}^{-T}\;{\left[\sum_{\ell =1}^{L}{\left({\hat{C}}_{k}^{\left(\ell \right)}\right)}^{-1}\right]}^{-T}=$$

$${\left[\sum_{\ell =1}^{L}{\left({\hat{C}}_{k}^{\left(\ell \right)}\right)}^{-1}\right]}^{-1}\sum_{i=1}^{L}{\left({\hat{C}}_{k}^{\left(i\right)}\right)}^{-1}{\hat{C}}_{k}^{\left(i\right)}\;{\left({\hat{C}}_{k}^{\left(i\right)}\right)}^{-T}\;{\left[\sum_{\ell =1}^{L}{\left({\hat{C}}_{k}^{\left(\ell \right)}\right)}^{-1}\right]}^{-T}$$

$$={\left[\sum_{\ell =1}^{L}{\left({\hat{C}}_{k}^{\left(\ell \right)}\right)}^{-1}\right]}^{-1}\sum_{i=1}^{L}{\left({\hat{C}}_{k}^{\left(i\right)}\right)}^{-T}\;{\left[\sum_{\ell =1}^{L}{\left({\hat{C}}_{k}^{\left(\ell \right)}\right)}^{-1}\right]}^{-T}={\left[\sum_{\ell =1}^{L}{\left({\hat{C}}_{k}^{\left(\ell \right)}\right)}^{-1}\right]}^{-1}$$

Therefore,

(A1) $${\hat{C}}_{k}^{F}={\left[\sum_{\ell =1}^{L}{\left({\hat{C}}_{k}^{\left(\ell \right)}\right)}^{-1}\right]}^{-1}$$

Now, we claim that

(A2) $${\hat{C}}_{k}^{F}⪯{\hat{C}}_{k}^{\left(\ell \right)}\;\mathrm{for}\;\mathrm{all}\;\ell$$

We know that

(A3) $${\left({\hat{C}}_{k}^{\left(i\right)}\right)}^{-1}⪰0,\;\;\;i=1,\ldots ,L$$

Therefore,

(A4) $$\sum_{i=1,i\neq \ell }^{L}{\left({\hat{C}}_{k}^{\left(i\right)}\right)}^{-1}⪰0$$

and

(A5) $${\left({\hat{C}}_{k}^{\left(\ell \right)}\right)}^{-1}+\sum_{i=1,i\neq \ell }^{L}{\left({\hat{C}}_{k}^{\left(i\right)}\right)}^{-1}⪰{\left({\hat{C}}_{k}^{\left(\ell \right)}\right)}^{-1}$$

which means that

(A6) $$\sum_{i=1}^{L}{\left({\hat{C}}_{k}^{\left(i\right)}\right)}^{-1}⪰{\left({\hat{C}}_{k}^{\left(\ell \right)}\right)}^{-1}$$

Therefore,

(A7) $${\left[\sum_{i=1}^{L}{\left({\hat{C}}_{k}^{\left(i\right)}\right)}^{-1}\right]}^{-1}⪯{\left[{\left({\hat{C}}_{k}^{\left(\ell \right)}\right)}^{-1}\right]}^{-1}$$

and so

(A8) $${\hat{C}}_{k}^{F}⪯{\hat{C}}_{k}^{\left(\ell \right)}$$
